# Scraps

**Published:** 1901-06

**Authors:** 


### SCRAPS

PICKED UP BY L. M. G.

**Clinical Records** (35).—*The London Hospital Gazette* has a poem entitled "H. Brown, M.D.," by Herbert Crosse, in which occurs the following verse:—

An almost Scottish brusquerie
Adorned his personality,
So very literal was he
  E'en idiom he 'd debar you.
A duchess in her languid way,
Quoth she "I 'm not myself to-day."
"Good gracious, ma'am," he 'd bellow, "Pray!
  Then who the devil *are* you!"

**"The English as he is Printed."**—At the Restaurant Baleste, opposite La Hume Station, where Arcachon golfers leave their clubs, a correspondent of *The Traveller*, whilst sipping his quinquina, found that the label on the bottle read thus:—

"NOTICE.

"The quinquina at the coca and kola of the Doctor Cruzel is the most tractable and the most certain arms at defend the healty against the illness, or the maintenance of his integrity. The happy preparation of the Doctcr Cruzel asserts in obtaining the agreeableness of the savour, puttinez up quickness of all active principles of the quinquina withant to admit those which are contained in the coca, and whole together form the best the first from all the tonics.

"The quinquina is known, acts indeed like bitter, in the first digestive conveyances it is *eupeptic, ahaleptic*, it is a tonic of the stomach, one matchless auxiliary of the nutrition in distress; but it is especially a energetic modification nutritious, depth of activity and intimate of the organs. It is a stimulate, rebuild again, and regenerator tonic.

"It is the most astringent, anather way, tonic action it is at last antisptic and in favour to this invaluable quality it possess one incontestable efficacy in numerous septical affections, the sickness to microbe.

"All the stomachs cannot adapt the same wine, this quinquina is delivered at the consommation prepared with white or black wine in the manner to fit the wishes of whole consumers."

**The True Business of the Doctor.**—"There is one part of their business that certain medical practitioners are too apt to forget,—namely, that what they should most of all try to do is to ward off disease, to alleviate suffering, to preserve life, or at least to prolong it if possible. It is not of the slightest interest to the patient to know whether three or three and a quarter of his lungs are hepatized. His mind is not occupied with thinking of the curious problems which are to be solved by his own autopsy, whether this or that strand of the spinal marrow is the seat of this or that form of spinal degeneration. He wants something to relieve his pain, to mitigate the anguish of dyspnea, to bring back motion and sensibility to the dead limb, to still the tortures of neuralgia. What is it to him that you can localize and name by some uncouth term the disease which you could not prevent, and which you cannot cure? An old woman who knows how to make a poultice and how to put it on, and does it *tuto, cito, jucunde*, just when and where it is wanted, is better—a thousand times better in many cases—than a staring pathologist who explores and thumps, and doubts and guesses, and tells his patient he will be better to-morrow, and so goes home to tumble his books over and make out a diagnosis."—Oliver Wendell Holmes, quoted in *University Medical Magazine*.

**Medical Philology** (XXXVIII.).—In a note on "Chitterlings" which I gave in the *Journal* for March, 1896, reference was made to its connection with "haggis." Further matter of interest about this word is to be found in Mr. Herrtage's note on the *Catholicon* "a Dregbaly; *Aqualicul*us, *porci* est *ventripoten*s," which is as follows:—

'*Aqualiculus, Ventriculus, sed proprie porcorum pinguedo super umbilicum.*' Ducange. '*Ventriculus.* The stomacke. *Aqualiculus.* A parte of the belly; a paunche.' Cooper. Baret has also 'a Panch, *Rumen Aqualiculus.* A panch, or gorbellie guts, A tunbellie. *Ventrosus, ventricosus.*' '*Aqualiculus: ventriculus porci.*' Medulla. Perhaps the meaning here is the dish 'haggis.' The Ortus Vocabulorum gives '*Omasus, i e., tripa vel ventriculus qui continet alia viscera.* A trype, or a podynge, or a wesaunt, or hagges:' and Cotgrave has '*Gogue.* A sheepes paunch, and thence a haggas made of good herbes, chopt lard, spices, eggs, and cheese, the which incorporated and moistened with the warme blood of the (new-killed) beast, are put into her paunch, and sodden with other meat.' Withals says '*Ilia porcorum bona sunt, mala reliquorum.* The intrals of Hogges are good (I thinke he meaneth that which wee commonly call Hogges-Harslet).'

Under "Hagas," one of the many forms of "Haggis," Mr. Herrtage quotes "'*Tucetum*. A puddyng or an hakeys. *Tucetarius*. A puddyng makere.' Medulla. 'A haggesse, *tucetum*.' Manip. Vocab." The quotations in the *New English Dictionary* show that although "now considered specially Scotch," haggis was "a popular dish in English cookery down to the beginning of the 18th century." I gave details of its composition in my note on Chitterlings. In a Latin and English Vocabulary of the 15th century (Wright's *Vocab*. ed. Wülcker, 567, 2) occurs "*Aumentum*, *ance* an hagase."

"Dregbaly" is supposed by the *New English Dictionary* to be an error for "dragbelly," signifying "a person with a large paunch," but no instance for such a word has been found.

"**The Apothecary Poet.**"—From the "Memoir" in *The Complete Works of John Keats*, edited by Mr. H. Buxton Forman, and just published in an excellent and inexpensive form by Messrs. Gowans and Gray, I extract some passages in reference to Keats's illness, which form a pathetic addendum to the note I gave in the *Journal* for September, 1900.

On the 3rd of February, 1820, Keats's mortal illness declared itself. Brown records that on that night his friend came into the house "in a state that looked like fierce intoxication. Such a state in him," says Brown, "I knew, was impossible; it therefore was the more fearful.' I asked hurriedly, 'What is the matter? You are fevered.' 'Yes, yes,' he answered; 'I was on the outside of the stage this bitter day till I was severely chilled, but now I don't feel it. Fevered?—of course a little.' He mildly and instantly yielded—a property in his nature towards any friend—to my request that he should go to bed. I followed with the best immediate remedy in my power. I entered his chamber as he leapt into bed. On entering the cold sheets, before his head was on the pillow, he slightly coughed, and I heard him say, 'This is blood from my mouth.' I went towards him; he was examining a single drop of blood upon the sheet. 'Bring me the candle, Brown, and let me see this blood.' After regarding it steadfastly, he looked up in my face, with a calmness of countenance that I can never forget, and said, 'I know the colour of that blood; it is arterial blood—I cannot be deceived in that colour. That drop of blood is my death-warrant; I must die.' I ran for a surgeon; my friend was bled; and, at five in the morning, I left him after he had been some time in a quiet sleep." Fanny Brawne's account of the actual commencement of this mortal illness, though less picturesque and circumstantial, has a distinct value. She says it "began from inflammation in the lungs, from cold," and adds—"In coughing, he ruptured a blood-vessel. An hereditary tendency to consumption was aggravated by the excessive susceptibility of his temperament, for I never see those lines of Dryden without thinking how exactly they applied to Keats:—

> The fiery soul, that working out its way,
> Fretted the pigmy body to decay.

From the commencement of his malady he was forbidden to write a line of poetry, and his failing health, joined to the uncertainty of his prospects, often threw him into deep melancholy." After fresh attacks of bleeding from the lungs on the 22nd and 23rd of June, 1820, the doctors told him that another winter in England would be fatal; and here was a fresh and terrible complication. No wonder that when John and Maria Gisborne, as recorded in her manuscript journal, drank tea at Hunt's on the 12th of July, the glory had departed. "I was much pained," she wrote, "by the sight of poor Keats, under sentence of death from Dr. Lamb. He never spoke and looks emaciated." By the middle of November he was in Rome and settled in a lodging in the Piazza di Spagna, in the first house on the right-hand side as one ascends the beautiful stairway of the Trinità dei Monti. Repeated attacks of hæmoptysis brought about the end on February 23, 1821. Severn, who was with him, says, in a letter on the 27th; "He is gone; he died with the most perfect ease—he seemed to go to sleep. On the 23rd, about four, the approaches of death came on. 'Severn—I—lift me up—I am dying—I shall die easy; don't be frightened—be firm, and thank God it has come.' I lifted him in my arms. The phlegm seemed boiling in his throat, and increased until eleven, then he gradually sunk into death, so quiet, that I still thought he slept."

